# The Phoenix of stem cells: pluripotent cells in adult tissues and peripheral blood

**DOI:** 10.3389/fbioe.2024.1414156

**Published:** 2024-07-30

**Authors:** Ranieri Cancedda, Maddalena Mastrogiacomo

**Affiliations:** ^1^ Dipartimento di Medicina Sperimentale, Università degli Studi di Genova, Genova, Italy; ^2^ Dipartimento di Medicina Interna e Specialità Mediche (DIMI), Università Degli Studi di Genova, IRCCS Ospedale Policlinico San Martino, Genova, Italy

**Keywords:** muse cells, very small embryonic like cells, VSELs, mesenchymal stem cells, dedifferentiation, pluripotent genes, SSEA3/4

## Abstract

Pluripotent stem cells are defined as cells that can generate cells of lineages from all three germ layers, ectoderm, mesoderm, and endoderm. On the contrary, unipotent and multipotent stem cells develop into one or more cell types respectively, but their differentiation is limited to the cells present in the tissue of origin or, at most, from the same germ layer. Multipotent and unipotent stem cells have been isolated from a variety of adult tissues, Instead, the presence in adult tissues of pluripotent stem cells is a very debated issue. In the early embryos, all cells are pluripotent. In mammalians, after birth, pluripotent cells are maintained in the bone-marrow and possibly in gonads. In fact, pluripotent cells were isolated from marrow aspirates and cord blood and from cultured bone-marrow stromal cells (MSCs). Only in few cases, pluripotent cells were isolated from other tissues. In addition to have the potential to differentiate toward lineages derived from all three germ layers, the isolated pluripotent cells shared other properties, including the expression of cell surface stage specific embryonic antigen (SSEA) and of transcription factors active in the early embryos, but they were variously described and named. However, it is likely that they are part of the same cell population and that observed diversities were the results of different isolation and expansion strategies. Adult pluripotent stem cells are quiescent and self-renew at very low rate. They are maintained in that state under the influence of the “niche” inside which they are located. Any tissue damage causes the release in the blood of inflammatory cytokines and molecules that activate the stem cells and their mobilization and homing in the injured tissue. The inflammatory response could also determine the dedifferentiation of mature cells and their reversion to a progenitor stage and at the same time stimulate the progenitors to proliferate and differentiate to replace the damaged cells. In this review we rate articles reporting isolation and characterization of tissue resident pluripotent cells. In the attempt to reconcile observations made by different authors, we propose a unifying picture that could represent a starting point for future experiments.

## Introduction

A pluripotent stem cells is defined as a stem cell from which cells of lineages from all three germ layers, ectoderm, mesoderm, and endoderm, can be derived. Embryonic Stem Cell (ESCs) isolated from the inner cell mass of a blastocyst, an early-stage pre-implantation embryo, are an example of pluripotent stem cells. On the contrary, multipotent and unipotent stem cells can develop into more than one or one cell type respectively, but their differentiation is limited to the cell types found in the tissue of origin or, at most, from the same germ layer. Multipotent adult stem cells have been isolated from a variety of adult tissues, including bone-marrow and cord blood. Instead, the presence of pluripotent stem cells in adult tissues and in peripheral blood has been, and still is, a very debated issue among scientists.

In October 2012, the Nobel Prize in Physiology or Medicine was awarded to two scientists in the field of cell reprogramming, Sir John Gurdon of the United Kingdom, and Shinya Yamanaka of Japan. John Gurdon was awarded for his seminal experiment demonstrating that the nucleus of a mature somatic cell transplanted into an enucleated frog’s egg, was reprogrammed to an embryonic-like state able to develop into a cloned frog. Shinya Yamanaka showed the reversion of a terminally differentiated cell to a state like an embryonic stem cell through the transfection with four genes expressed in early embryo cells, namely *Oct3/4, Sox2, Klf4,* and *c-Myc*, (induced Pluripotent Stem Cells – iPSCs). This has been one of more exiting discovery of the biology in the recent years and opened great therapeutic perspectives for many diseases and patients. However, the generation of pluripotent cells by the transfection of genes expressed in the early embryo will be not considered and discussed in this review.

The review interest is in adult tissue resident stem cells and in cell physiological dedifferentiation that occurs spontaneously *in vivo* or after a minimal manipulation *in vitro*. However, given that sometimes Hemopoietic Stem Cells (HSCs) and Mesenchymal Stem Cells (MSCs), for their capacity to give rise to different types of differentiated cells, were erroneously considered pluripotent cells, and that it was also suggested that these cell populations include few pluripotent cells present in very low concentrations, in the initial two paragraphs we discuss HSCs and MSCs and we correctly define them multipotent cells. Moreover, HSCs are the archetypes of stem cells and the studies aimed at the definition of their nature and functions set the ground for the subsequent investigations on pluripotent stem cells.

The concept that adult stem cells are restricted to their own tissue has been questioned by the work of several labs indicating that stem/progenitor cells can be found also in the peripheral blood in response to a tissue damage. It has been also reported that circulating cells play a crucial role in the organ repair either by directly implanting in the damaged tissue and generating progenitors and new differentiated cells or by enhancing a re-activation of resident stem cells (Cooke, 2019).

## Hemopoietic stem cells (HSCs) and endothelial progenitors (EPs), archetypes of adult stem cells, are multipotent but not pluripotent cells

Hemopoietic Stem Cells (HSCs) were the first stem cells identified in the bone-marrow of mammals (mice and humans). These stem cells are also isolated from the peripheral blood in physiological steady-state conditions (Bourdieu et al., 2018; Lapostolle et al., 2018). Their concentration in the peripheral blood is enhanced after mobilization following a Granulocyte Stimulating Factor treatment (Thomas et al., 2002). These studies leaded not only to the identification of the HSCs, comprising both long-term and short-term HSCs, but also to the identification of the Hemangioblast, as progenitor of both hemopoietic and endothelial cell lineages. However, both hemoangioblasts and HSCs should be defined as multipotent stem cells and cannot be considered pluripotent since their progeny is limited to tissues of mesodermal origin.

After the atomic bomb explosions and the end of World War II, several scientists started to investigate the effect of X ray radiations on leukemia onset in exposed subjects. In the sixties, in the context of their study on the minimal bone marrow cell number exhibiting a “resuscitating” effect on mice after total body lethal-dose irradiation, Till and McCulloch and their coworkers discovered Colony Forming Units (CFUs), established that CFUs arises from a single cell, and published a series of articles reporting that i) in the mice bone-marrow CFUs could give rise to mixed myeloerythroid progeny (granulocytes, macrophages, red cells, megakaryocytes), ii) some of these cells duplicate themselves, iii) in the spleens of mice, cells existed that could also make lymphocytes. More important, Till and McCulloch showed that a bone-marrow transplant could restore blood cell formation and survival in mice whose fate was death because of an impairment in blood cell production (Becker et al., 1963; Siminovitch et al., 1963; Wu et al., 1967; Wu et al., 1968; Thomas et al., 2002).

Most mature blood cells are short lived, and HSCs must continuously provide differentiating progenitors and, at the same time, maintain the HSC pool throughout the whole individual life. Two types of HSCs have been identified: long-term HSCs which are a rare, quiescent population in bone marrow with a more than 3∼4 months capacity of self-renewal, and short-term HSCs which do not. Short-term HSCs have limited self-renewal capacity, but, for 4–12 weeks before senescence, give rise to committed progenitors and precursor cells leading to all blood cell types, characterized by specific markers. For a review (Seita and Weissman, 2010).

HSCs are commonly characterized by the absence of lineage-specific marker expression and the presence of specific cell surface antigens. Human Hemopoietic Stem Cells are CD34^+^, CD59^+^, CD90/Thy1^+^, CD38^low/−^, c-Kit^−/low^, and Lin^−^. Mouse Hemopoietic Stem Cells are CD34^low/−^, SCA-1^+^, CD90/Thy1^+/low^, CD38^+^, c-KIT^+^, and LIN^−^. To isolate most primitive long-term HSCs, Christensen and Weissman sorted highly purified multipotent stem and progenitor cells based on the Flk-2/Flt3 receptor tyrosine kinase surface expression. The low number of Flk-2− HSCs gave rise to long-term multilineage reconstitution in the majority of recipients, whereas the isolation of the Flk-2+ prevalent cell population resulted in mostly short-term multilineage stem cell formation (Christensen and Weissman, 2001).

Following the pioneer work of Till and Mc Cullogh the first successful bone-marrow transplant was performed in 1956 by by E. Donnall Thomas, Nobel prize for Physiology or Medicine in 1990 (Thomas et al., 1957). A child with leukemia received a transplant with bone-marrow from the identical twin. In 1958 George Mathè, after a nuclear accident, performed the first allogeneic transplant on six patients at the Institute Curie in Paris [cited by Jansen (2005)]. Since then, thousands of patients with leukemia or immunological deficits beneficed of this therapy. More recently, it was found that administration of Granulocyte Colony-Stimulating Factor (G-CSF) alone or combined with Plerixafor, a molecule which reversibly inhibits the binding of Stromal-Derived Factor-1 (SDF-1) to CXCR4, promotes HSC mobilization from bone-marrow to the peripheral blood. The mobilized HSCs can be collected, frozen, and stored until the time of transplant. The detailed mechanisms of the G-CSF-mediated HSC mobilization remain to be elucidated. Currently, the infusion of peripheric blood HSCs originated from the bone-marrow replaced the traditional bone-marrow aspirate transplant in most pathological conditions requiring an HSC transplant.

Given the spatial and temporal closeness of the outcome of blood vessels and red blood cells during embryogenesis, the existence of a common progenitor for the hemopoietic and the endothelial lineages was hypothesized by several hematologists already in the first half of last century. However, only in 1997, Kennedy et al. first isolated an *in vitro* equivalent of the hemangioblast. They plated aggregates of differentiating mouse embryonic stem cells (embryoid bodies) just before the arise of hemopoietic cells and in the presence of the appropriate cytokines. A subset of these cells was able to differentiate into both hemopoietic lineages (Kennedy et al., 1997) and endothelial cells (Choi et al., 1998). Few years later, a definitive proof of the same clonal origin of mouse blood and endothelial cells was obtained by the same group of authors using limiting dilution cultures of the mesoderm in the gastrulating embryo (Huber et al., 2004). Interestingly, some of the G-CSF mobilized cells give rise to differentiated cells of both hemopoietic and endothelial cell lineages (Loges et al., 2004).

For long time it was assumed that once the hemopoietic cells enter the pathway to mature blood cells, they cannot revert to an earlier differentiation stage. This belief was, often uncritically, extended to other types of stem cells subsequently discovered. More recent data cast some doubts on this belief especially for stem cells other than HSCs.

## Multi-lineage differentiating stress enduring cells (Muse cells) can be isolated from cultures of mesenchymal stem cells (MSCs)

More than 50 years ago, Friedenstein et al. isolated from bone-marrow a progenitor of skeletal tissues in the form of a colony-forming unit (CFU-F) (Friedenstein et al., 1970). These cells were renamed Mesenchymal Stem Cells (MSCs) in the ’80 of last century by Arnold Caplan (Caplan, 1991) although they do not have all the characteristic properties of a stem cell [see a recent editorial on the MSC terminological issue (Ivanovic, 2023)]. MSCs are a very heterogeneous population that became very popular in the tissue engineering and regenerative medicine community for their capacity to differentiate into tissues of mesodermal origin such as cartilage, bone and adipose. MSCs has been and is adopted for successful repair and regeneration of these wounded tissues but cannot be considered pluripotent given the differentiation to tissues of only mesodermal origin. Nevertheless, some rare report claiming that in certain conditions MSC could also differentiate into tissues of ectodermal and endodermal origin are present in the literature. Apparently, a very rare sub-population of MSC has the capacity to self-renew, and to differentiate into cells representative of all three germ layers from a single cell. This subpopulation, named Multi-lineage differentiating stress enduring cells (Muse cells), was first isolated and described in 2010 by Kuroda et al. from bone marrow stromal cells, or directly from bone marrow aspirates and dermal fibroblasts (Kuroda et al., 2010). Muse cells self-renew and express a set of genes associated with pluripotency; They were isolated by cell sorting as stage specific embryonic antigen-3 (SSEA-3) and CD105 double-positive cells. Alternatively, they were selected and isolated as cells resisting to stressful culture conditions. Muse cells have relatively low proliferation activity, and form characteristic cell clusters in suspension culture, like embryoid bodies of ES cells. As ES cells, Muse cells express pluripotent genes such as *Nanog, Oct 3/4, Sox 2,* and *TRA1-60.* At variance with ES cells, Muse cells are not teratogenic. Basic expression level of pluripotency genes in non-Muse cells is very low or undetectable compared to Muse cells. When human MSC were separated into Muse and non-Muse cells and transduced with *Oct3/4, Sox2, Klf4,* and *c-Myc*, iPS cells were generated exclusively from Muse cells but not from non-Muse cells (Wakao et al., 2011). This finding seems to support the elite versus the stochastic model for the generation of iPS cells. For reviews on this subject see (Kitada et al., 2012; Lv et al., 2014). When transplanted into immunodeficient mice by local or i.v. injection, Muse cells integrated into wounded tissues and spontaneously differentiated into the tissue specific cells (Kuroda et al., 2010). Non-Muse cells did not exhibit tissue reparation when infused into the blood stream. Although they do not integrate into the damaged tissue, non-Muse cells may indirectly promote tissue regeneration by their production of cytokines, trophic factors, and anti-inflammatory factors.

A small population of pluripotent stem cells with the same characteristic of bone-marrow derived Muse cells were obtained from adult human adipose tissue, cultures of adipose-derived Mesenchymal Stem Cells (adipose-Muse cells) (Heneidi et al., 2013) and also from cultures of human MSC derived from connective tissues like dermis (Kuroda et al., 2010; Wakao et al., 2011). As bone-marrow Muse cells, adipose-Muse cells are positive for MSC markers CD105 and CD90 and for the human pluripotent stem cell marker SSEA-3. They intrinsically retain lineage plasticity and the ability to self-renew and can generate cells representative of all three germ layers from a single cell. To note that adipose-Muse cells present low telomerase activity. Non-Muse adipose-MSC do not have the same properties and cannot cross the boundaries from mesodermal to ectodermal or endodermal lineages even with cytokine inductions. When compared with bone marrow (BM)- and dermal-Muse cells, adipose-Muse cells had the tendency to express higher levels of mesodermal lineage markers, whereas BM- and dermal-Muse cells expressed higher levels of markers of ectodermal and endodermal lineages. This suggests the existence of some influence on the cell differentiation potential by the microenvironment of the tissue from which Muse cells were isolated.

## Muse cells for regenerative medicine

Given their lack of tumorigenesis, the homing and integration in the damaged tissues, and the non-engraftment in organs and tissues other than the damaged ones, the Muse cells appeared since their discovery as almost ideal cells to be adopted for regenerative medicine. In the recent years several preclinical trials confirmed the regenerative potential of Muse cells.

With few exceptions, in all preclinical studies, Muse cells were of human bone-marrow origin and the recipient animals were immunocompromised mice with different pathologies. However, in some studies rat and rabbits were utilized as recipient animals. In most preclinical trials, the Muse cell injection was significantly beneficial. In some cases, in addition to the Muse cell integration and differentiation into cells of the damaged tissues, it was reported also a significant positive cell paracrine activity. A selection of different animal models of human diseases that were treated with injection of Muse cells are listed in Table 1. Following the preclinical trials, recently, a Japanese team published the results of a human single-center open phase II clinical trial to evaluate the safety and efficacy of repeated intravenous injections of Muse cells in patients with Amyotrophic lateral sclerosis (ALS), a neurodegenerative disease characterized by a progressive loss of motor functions. Although the trial main goal was to show the safety of treatments, they also observed that a monthly injection for 6 months could prevent or delay the symptoms of ALS from worsening (Yamashita et al., 2023).

**TABLE 1:** Animal models of human diseases treated with injection of Muse cells.

Disease	Muse origin			Muse role		References
		human	animal	paracrine	direct	
Heart infarct	Bone-marrow	x	x		x	Yamada et al. (2018)
Aortic aneurism	Bone-marrow	x			x	Hosoyama et al. (2018)
Lung ischemia	Bone-marrow	x		x	x	Yabuki et al. (2018)
Liver fibrosis	Bone-marrow	x			x	Iseki et al. (2017)
Hepatectomy	Bone-marrow	x			x	Katagiri et al. (2016)
Gastro-intestinal radio syndrome	Wharton’s jelly	x		x		Dushime et al. (2023)
Kidney diseases	Bone-marrow	x		x	x	Uchida et al. (2018)
Bladder inflammation	Bone-marrow	x		x	x	Furuta et al. (2022)
Spinal cord injury	Bone-marrow	x			x	Kajitani et al. (2021)
Spinal cord injury	Bone-marrow	x			x	Takahashi et al. (2023)
Spinal cord injury	Umbilical cord	x			x	Leng et al. (2019)
Hind limb ischemia	Bone-marrow	x		x		Hori et al. (2022)
Brain stroke	Bone-marrow	x			x	Yamauchi et al. (2015)
Brain stroke	Bone-marrow	x		x	x	Abe et al. (2020)
Brain stroke	Dermis	x			x	Uchida et al. (2016)
Brain hemorrhage	Bone-marrow	x			x	Shimamura et al. (2017)
Encephalopathy by *E. Coli*	Bone-marrow	x		x	x	Ozuru et al. (2020)

Other human clinical studies testing the efficacy of the intravenous injection of allogenic bone-marrow Muse cells are currently being performed in Japan for myocardial infarct (JAPIC ID: JapicCTI-183834 and JAPIC ID: JapicCTI-195067), stroke (JAPIC ID: JapicCTI-184103), spinal cord injury (JAPIC ID: JapicCTI-194841), and epidermolysis bullosa (. JAPIC ID: JapicCTI-184563.). These studies involve the injection of allogenic cells without the need of human leukocyte antigen-matching or long-term immunosuppression because human Muse cells possess immunomodulatory abilities allowing them to evade host immune cells at the injury site (Park et al., 2021).

## Dedifferentiated adipose-derived cells (DFATs) derived from mature adipocytes and adipose-MSCs

**FIGURE 1:**
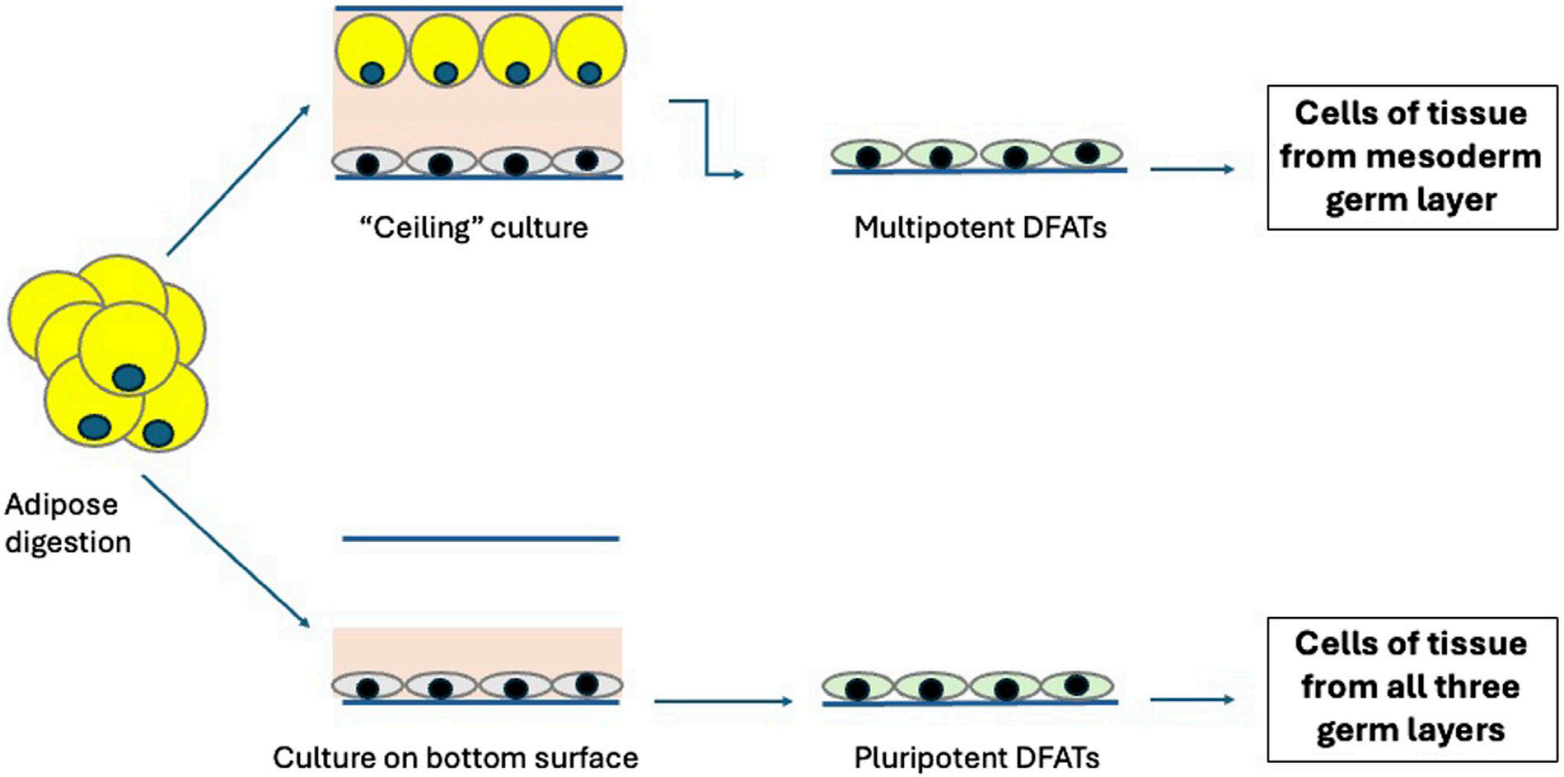
Generation of dedifferentiated adipose-derived cells (DFATs). Multipotent or pluripotent dedifferentiated cells are obtained depending on the culture conditions. Multipotent cells are derived from purified adipocytes isolated by “ceiling” culture. Pluripotent cells are obtained by cultures of bulk cells obtained by adipose digestion.

Some authors reported that in addition to Muse cells a new type of stem cells can be obtained from adipose and named this population Dedifferentiated adipose-derived cells (DFATs) (Miyazaki et al., 2005). Significative differences in the cell properties were observed depending upon the method of preparing DFAT cells. ‘‘Ceiling culture’’ is a method of culturing fat cells by allowing mature adipocytes in the floating layer to attach to the ceiling surface of culture flask filled with medium. Mature adipocytes isolated as a pure cell population by “ceiling,” when replated, attached, changed their morphology into fibroblastic cells proliferating as a monolayer (Figure 1) (Sugihara et al., 1986). DFATs obtained with this isolation and dedifferentiation protocol maintained a memory of their mesodermal origin, had a reprogramming ability which was associated to their dedifferentiation, and differentiated *in vitro* toward adipogenic, osteogenic, and chondrogenic lineages (Miyazaki et al., 2005). When the method of preparing DFAT cells did not include attachment of the adipocytes to ceiling plastic surfaces, as above described, the cells were directly expanded as monolayer and subjected to an *in vitro* dedifferentiation - redifferentiation protocol, they reverted to a primitive phenotype. These DFATS cells spontaneously formed clusters in culture, gained better differentiative capacities, and transiently expressed multiple stem cell markers, including stage specific embryonic antigens, and Sca-1 (mouse) and CD105 (human). As the embryonic markers decreased, markers characteristic of specific lineages of the three germ layers increased. Again, no teratoma formation was detected after injection in immunodeficient mice (Jumabay and Boström, 2015). DFATS presented on the surface markers for CD13, CD29, CD44, CD90, CD105, CD9, CD166 and CD54, and do not express CD14, CD31, CD34, CD45, CD66b, CD106, CD117, CD133, CD146, CD271, CD309, HLA-DR and alpha-smooth muscle cell actin; a fraction of DFATs also expressed SSEA-3 (Jumabay et al., 2014).

It is very likely that DFATs obtained from adipocytes cultured as traditional monolayer culture without a ceiling culture passage are the same cell population then adipose-derived Muse cells isolated from cultured adipose MSCs, and that the small diversities observed are the results of some differences in the protocols for cell isolation and expansion. Adipose-derived Muse cells also spontaneously generated cells of all three germ layers from a single cell and evolved to terminally differentiated cells after cytokine induction. Moreover, adipose-Muse cells had low telomerase activity and their transplants did not promote teratogenesis or tumorigenesis *in vivo* (Ogura et al., 2014). Adipose-derived MSCs were introduced as an alternative to bone-marrow MSCs for cell-based therapy. On the contrary, DFATs obtained from adipocytes isolated by the “ceiling” culture cannot be considered like Muse cells, and possibly are a lineage intermediate of this population since their redifferentiation is restricted to cells of mesoderm origin.

## Multipotent adult progenitor cells (MAPCs)

Multipotent adult progenitor cells (MAPCs) are bone marrow-derived non-hemopoietic stem cells with a broad differentiation potential and extensive expansion capacity. They were isolated from human, mouse, and rat postnatal bone-marrow. MAPCs, could be expanded *in vitro* maintaining an undifferentiated state for more than 100 population doublings, and could be differentiated into cells with morphological, phenotypic, and functional characteristics of mesodermal and neuroectodermal cells *in vitro* and into all embryonic lineages *in vivo* (Schwartz et al., 2002). At variance with other described populations of adult pluripotent cells, in most cases of human origin, it was possible to show that a murine single MAPC can integrate in the blastocysts giving rise to chimeras (Keene et al., 2003). MAPCs, cultured on Matrigel with FGF-4 and HGF, also differentiated into epithelioid cells expressing hepatocyte markers and acquired functional characteristics of hepatocytes. They secreted urea and albumin, had phenobarbital-inducible cytochrome p450, could take up LDL, and stored glycogen. A comparative study between human mesenchymal stem cells (hMSCs) and human MAPCs (hMAPCs) showed that hMAPCs have clearly distinct phenotypical and functional characteristics from hMSC. hMAPCs express lower levels of MHC class I than hMSCs and do not have a differentiation potential restricted to mesodermal lineages.

Stem cells with some MAPC properties were isolated also from tissues other than bone-marrow. Although it is generally accepted that adult stem cells from other tissues have a restricted differentiation ability and only generate cells from the tissue from which they were derived, some studies suggested that under certain conditions, adult tissue derived MAPCs may have a broader differentiation potency (Sohni and Verfaillie, 2011).

Human MAPCs have potent immunomodulatory properties *in vitro* and are non-immunogenic for T-cell proliferation and cytokine production (Jacobs et al., 2013). MAPCs induce regulatory T cells and promote their suppressive phenotype via TGFβ and monocyte-dependent mechanisms (Valentin-Torres et al., 2021). They can serve as a valuable source of an immunomodulatory cellular product for the clinical use. The immunoregulatory capacity of MAPC cells was evaluated *in vivo* using established murine graft-versus-host disease (GVHD) models. Human MAPCs effectively reduced graft-vs-host disease while preserving graft-vs-leukemia (Metheny et al., 2021). A clinical grade human MAPC product is already used in clinical trials to prevent GVHD, as well as for the treatment of acute myocardial infarct, ischemic stroke, and Crohn’s disease. In the clinical trials, MAPCS are used not for their direct regenerative effect but for their secretion-based immunosuppressive effect. Moreover, MAPCs secrete a wide range of factors known to accelerate the wound healing process, and their secretome has a strong positive impact on healing outcomes without the need of MAPC cell presence (Ahangar et al., 2020).

## Marrow-isolated adult multilineage inducible (MIAMI) cells

The isolation of a unique subpopulation of human stromal cells from bone marrow termed marrow-isolated adult multilineage inducible (MIAMI) cells was reported by D’Ippolito et al. (2004). The expression of embryonic stem cell markers SSEA-4, Oct-4, Rex-1, and telomerase reverse transcriptase indicated the developmentally immature status of these cells. MIAMI cells are characterized by a unique molecular profile that distinguishes them from other marrow stromal cell populations. To isolate MIAMI cells from human bone marrow with a unique expansion/selection procedure, marrow-adherent and -nonadherent cells were co-cultured on fibronectin, at low oxygen tension, for 14 days (D’Ippolito et al., 2004). Since the oxygen concentration in bone marrow ranges from 1% to 7%, the role of oxygen tension in regulating the capacity of MIAMI cells to self-renew and to maintain the pluripotential was investigated. Low oxygen concentration during long-term culture upregulated the expression of mRNAs of pluripotent genes and markers confirming that a low oxygen tension is more physiologic for these cells and favors stemness (D’Ippolito et al., 2006). Cells of small colonies were expanded on fibronectin at low density, low oxygen concentration and 2% FCS. Cell-doubling was 36–72 h, and the number of doublings that the cells could perform in culture was more than 50. Expanded cells expressed the early embryo markers Oct-4 and Rex-1, had a telomerase activity, were negative for CD34, CD36, CD45, CD117 (cKit) and HLADR, and positive for CD29, CD63, CD81, CD122, CD164, hepatocyte growth factor receptor (cMet), bone morphogenetic protein receptor 1B (BMPR1B), and neurotrophic tyrosine kinase receptor 3 (NTRK3). MIAMI cells could be induced to differentiate, at least *in vitro*, into cells of tissue of mesodermal origin, such as cartilage, bone and adipose, as well as cells of tissues with an ectodermal or endodermal origin.

Additional studies investigated the therapeutic value of the MIAMI cells to induce neovascularization in a mouse model of critical hindlimb ischemia. Compared with animals receiving other types of cells, MIAMI cells significantly improved blood perfusion, and reduced necrosis and inflammation in the ischemic limb (Rahnemai-Azar et al., 2011). In a different preclinical trial, MIAMI cell-seeded on gelatin fibers leaded to an almost full recovery of blood flow in an injured mouse limb, thus limiting the extent of ischemia and necrosis. Histology was performed 28 days after grafting the animals. The use of MIAMI cells-seeded gelatin prevented intermuscular infiltration by adipose tissue and evidenced some muscular fibers regeneration (Grau-Monge et al., 2017). MIAMI cells injected into the hippocampus prevented neuronal damage induced by global ischemia in rat hippocampal slices exposed to oxygen-glucose deprivation and rats subjected to asphyxia cardiac arrest. Their therapeutic value was significantly increased when cells were delivered complexed with fibronectin-coated biomimetic microcarriers thus increasing stem cell survival and paracrine secretion of pro-survival and/or anti-inflammatory molecules (Garbayo et al., 2011). Interestingly, MIAMI cells were also used as cellular carriers of drug-loaded nanoparticles to brain tumors. MIAMI cells specifically migrated toward the orthotopic U87MG tumor model and did not influence its growth (Roger et al., 2012).

## Dedifferentiated cells as a potential source of stem cells

Dedifferentiation is a transient process by which cells become less specialized and return to an earlier cell state within the same lineage. Dedifferentiation of terminally differentiated cells and redifferentiation to generate a new individual is a process common to many vegetables. In animals, dedifferentiation and redifferentiation is relatively frequent in early embryos but after birth is maintained only in some invertebrates and in organs of lower vertebrates, such as newt and salamander limbs (Joven et al., 2019) and zebra fish heart (Jopling et al., 2010). In mammals, and in humans in particular, except for liver, dedifferentiation and redifferentiation has been observed mostly in *in vitro* cultured cells. The articular chondrocyte dedifferentiation and redifferentiation is an example of a mammal cell culture system well documented and deeply investigated. The differentiated phenotype of articular chondrocytes is characterized by the synthesis of type II collagen and cartilage-specific proteoglycan. When cultured in Petri dishes in the presence of MSC culture medium, chondrocytes acquire a flattened anchorage-dependent morphology and switch to the synthesis of type I collagen and of a low level of proteoglycan. As such, dedifferentiated chondrocytes can be passaged as monolayer cultures. However, when transferred to an anchorage-independent culture, the cells reacquire a spherical morphology, resume the synthesis of type II collagen and proteoglycans and returned to the differentiated phenotype, characteristic of primary chondrocytes (Benya and Shaffer, 1982). In the ’80 of last century, our research team showed that dedifferentiated endochondral chondrocytes with a fibroblastic morphology and expressing type I collagen, common to most connective tissues, when transferred in suspension culture, formed cell aggregates (spheroids), reverted to a differentiation pathway toward mature endochondral hypertrophic chondrocytes (Castagnola et al., 1986), halted the production of type I collagen, and expressed in sequence the cartilage specific collagens, type II and type X (Castagnola et al., 1988). Cell aggregates differentiating to hypertrophic chondrocytes were obtained in suspension cultures also from about 50% of cell populations derived from a single cloned dedifferentiated cell (Quarto et al., 1990). The 50% non-differentiating cell clones maintained the fibroblastic morphology and the expression of type I collagen.

The freeing from the pre-existing matrix and the cell adherence to the culture dish are crucial for chondrocyte dedifferentiation. Focal adhesion kinase (FAK) provides signaling at sites of integrin adhesion. FAK knockdown promoted the restoration of cartilage-specific gene expression in dedifferentiated chondrocytes (Shin et al., 2016). Also, pro-inflammatory cytokines, such as IL-1β, promoted the dedifferentiation of cultured chondrocytes. NF-kB pathway, a prototypical proinflammatory signaling pathway, by inducing pathways eventually leading to the activation of ERK and p38, enhanced the effect of IL-1β on the dedifferentiation of human chondrocytes *in vitro* (Li et al., 2019). For a more detailed information on molecular mechanisms controlling chondrocyte dedifferentiation see (Yao and Wang, 2020). The first surgical trial with autologous chondrocyte implantation (ACI) was performed in rabbit in 1989 (Grande et al., 1989) whereas the first pilot human study was published in 1994 (Brittberg et al., 1994). ACI was one of the first cell therapies approved by FDA and EMA regulatory agencies.

Other examples of cell dedifferentiation when transferred in culture after a minimal manipulation are epidermal keratinocytes, thyroid, peripheral nerves, pancreas islet cells.

i) Dedifferentiation of human epidermal keratinocytes into their precursor cells *in vitro* requires basic fibroblast growth factor (bFGF) but not external gene intervention (Sun et al., 2011). Following incubation with bFGF, few human terminally differentiated keratinocytes survived and reverted to a dedifferentiated state, as evidenced by re-expression of biological markers of native keratinocyte stem cells (KSCs), including β(1)-integrin, CK19 and CK14; ii) Multilineage progenitor cells were obtained from normal human thyroid tissues (Suzuki et al., 2011). To obtain dedifferentiated progenitor cells from tissue fragments, after enzymatic digestion, the primary thyrocytes, expressing thyroglobulin, vimentin, and cytokeratin-18, were cultured in a serum-free medium (called SAGM). Most cells died, but a small proportion (∼0.5%) survived and proliferated. During the initial cell expansion, thyroglobulin/cytokeratin-18 expression was gradually declined in the proliferating cells. The SAGM-expanded cells did not express thyroid-specific genes. However, after incubation with FBS and TSH, the cells re-expressed cytokeratin-18, thyroglobulin, TSH receptor, PAX8 and TTF1. Surprisingly, the cells could also differentiate into neuronal or adipogenic lineages depending on culture conditions; iii) Peripheral nerves regenerate spontaneously after injury because of a permissive environment and the activation of intrinsic growth capacity of neurons. Functional regeneration requires axon regrowth and remyelination by Schwann cells. Multiple factors, including neurotrophic factors, extracellular matrix (ECM) proteins, and hormones participate in Schwann cell dedifferentiation, proliferation, and remyelination (Chen et al., 2007); iv) Human islets embedded in a collagen gel after being exposed to EGF, dedifferentiated into duct-like epithelial structures (DLS). DLS formation was EGF dependent, while residual DLS formation in the absence of added EGF was abrogated by EGF receptor inhibitor treatment. EGF increased phosphorylation of c-Jun NH2-terminal kinase (JNK) early in DLS formation and of AKT and extracellular signal-regulated kinase (ERK) late in the process (Hanley et al., 2011). Thus, EGF is necessary for islet cell dedifferentiation, both at the onset of DLS formation (through JNK) and in the proliferation of these dedifferentiated cells (through AKT and ERK).

However, in all cited examples, dedifferentiated cells maintained the memory of the tissue of origin and did not have characteristics and properties of true pluripotent stem cells since they were not able to generate tissues derived from other germ layers. Therefore, it appears a general rule that, at most, dedifferentiated cells can be considered tissue specific multipotent stem cells and not *bona fide* pluripotent stem cells.

## Very small embryonic-like cells

### First identification by the Louisville research team

Very Small Embryonic-Like cells (VSELs) were identified by the research group of Mariusz Ratajczak at the University of Louisville, Kentucky, United States first in murine bone marrow (Kucia et al., 2006) and other murine organs and then in human umbilical cord blood, peripheral blood, and gonads (Kucia et al., 2008; Sovalat et al., 2011). VSELs are small cells lineage negative (lin^−^), do not express CD45 antigen (CD45^−^), display a high nuclear/cytoplasm ratio and express some antigens characteristic of early embryo cells. After the initial 2006–2008 publications, more than 40 articles expanding the initial observations have been published by the Ratajczak group. The main findings can be summarized as such: i) VSELs measure ∼3–5 μm in mice and ∼5–7 μm in humans and are purified trough a sophisticated fflow-cytometry gating protocol; ii) express embryonic stem cell markers, including stage specific embryonic antigen (SSEA), nuclear Oct-4A, Nanog, and Rex1; iii) originate from cells related to the germline, are deposited in developing organs during embryogenesis, and play a role as a backup population for non-pluripotent tissue-committed stem cells; iv) are quiescent in bone marrow and other tissues and organ but are activated during stress situations and mobilized into the circulation; v) are released into the peripheral blood after granulocyte colony-stimulating factor mobilization; vi) peripheral blood VSELs contain cell subpopulations committed along differentiation pathways leading to a variety of tissues across germ layers, such as hemopoiesis, osteogenesis, angiogenesis as well as myocardium, liver, and pulmonary alveolar epithelium; vii) the number of VSELs decreases with age. For a recent specific review see (Ratajczak et al., 2019).

**FIGURE 2:**
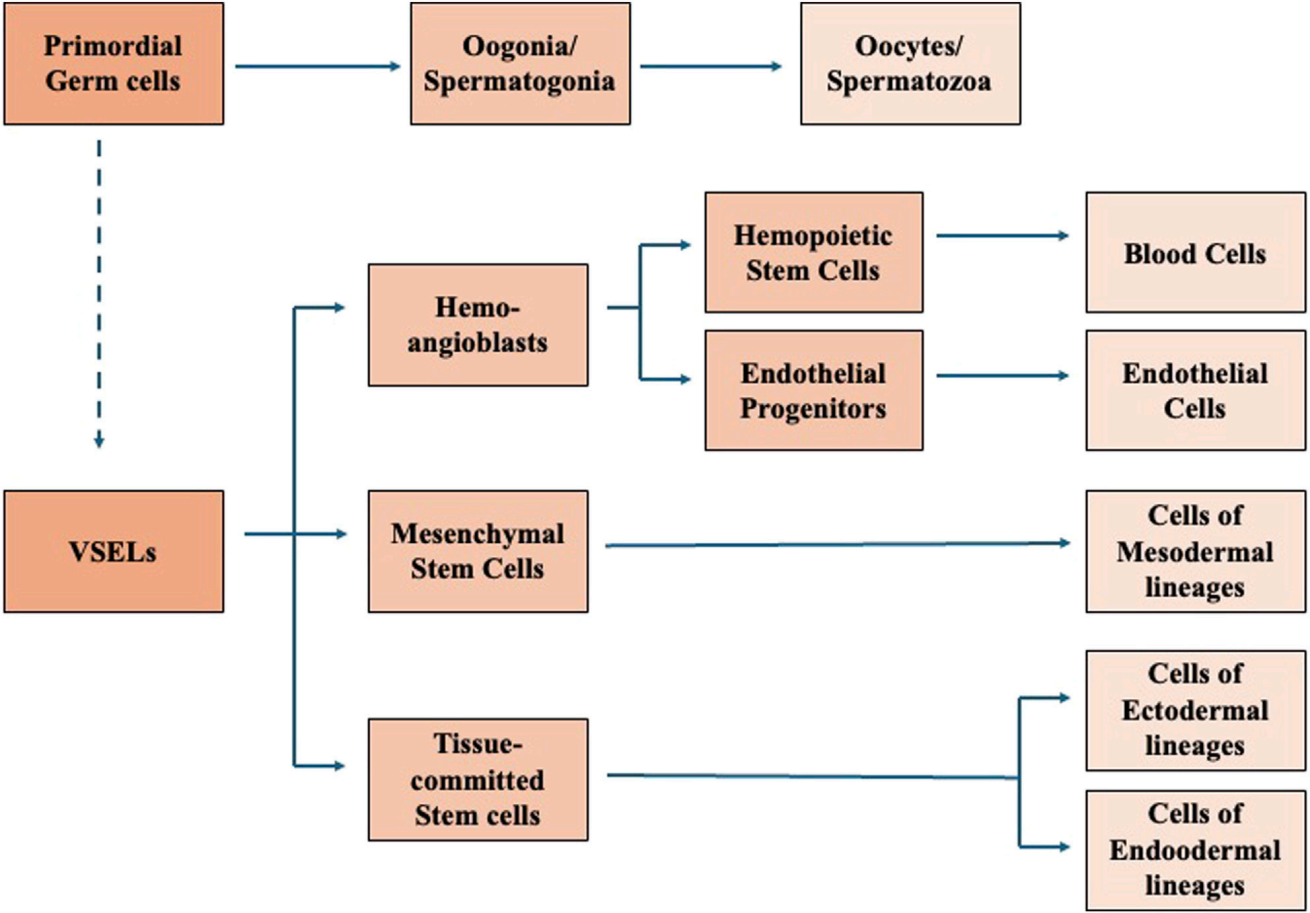
Proposed correlation between Primordial gem cells, VSELs, and VSEL progeny. Modified from Ratajczak et al. (2019).

Based on the above findings and other observations, Ratajczak et al. proposed that migratory primordial germ cells are the source of VSELs that in bone marrow give rise to hemopoietic stem cells, endothelial progenitors, and are a source of tissue-committed stem cells (Figure 2) (Ratajczak, 2017; Ratajczak et al., 2019). Moreover, based on some evidence that several adult tissues contain a population of cells expressing markers characteristic for embryonic stem cells, epiblast stem cells and primordial germ cells, such as stage specific embryonic antigen and transcription factors Oct-4 and Nanog, Ratajczak et al. suggested that also adult tissues contain some populations of pluripotent stem cells deposited in embryogenesis during early gastrulation (Ratajczak et al., 2007). However, alternative hypothesis could be made, and the pluripotent cells isolated from tissues could also be pluripotent stem cells migrated from the peripheral blood into the tissues in response to a local stimulus. Several papers showed a contribution by injected purified VSELs to hematopoiesis, osteogenesis, and angiogenesis, as well as to regeneration of myocardium, liver, and pulmonary alveolar epithelium in *in vivo* animal models. The observed chimerism in several organs after the injection confirmed the differentiation of VSELs across germ layers (Ratajczak et al., 2017).

Since the first identification of VSELs, the research brought to the Ratajczak group awards and prizes in recognition for their work, but also raised some concerns from the stem cell investigator community. Major criticisms were due to the initial inability to repeat the experiments by some major stem cell labs and to the fact that VSELs were since the beginning presented as ethical alternative research to prevent the further development of Embryonic Stem cells (ES cells) and induced pluripotent stem cells (iPS cells) and that the biotech company owing the 2011 Ratajczak patents received significant financial support by opponents of the use of human embryos. In addition, some reports and comments made on the web evidenced certain image duplications and redundancies and textual similarities in the published articles. Because of these allegations, in 2021 the University of Louisville made a deep investigation and found no research misconduct. Moreover, by now, other scientists have independently obtained similar results. Nevertheless, critic comments continue to be posted on the internet.

### Investigations by other research teams

Rare pluripotent cells of small size expressing genes characteristic of early embryo cells has been described also by other research teams independently from the Louisville team. In most cases, the authors maintained the VSELs name. Nakatsuka et al. developed a highly efficient method to isolate Lin(−), CD45 (−), Sca-1 (+) small cells by enzymatic digestion of murine bone. They designated these cells as bone derived VSELs (BD VSELs). In the steady state, the BD VSELs proliferated slowly, but their number significantly increased in the bone after an acute injury. Apparently, cells with the properties of VSELs are present in the blood of human embryos and fetuses. Several research groups, adopting different protocols, isolated, from human cord blood, cells of small size (3–6 μm), expressing pluripotent genes, such as Oct 4, Nanog, SSEA4 and Sox2, and with the potential to differentiate toward the meso-endo-ectoderm lineages, including neural differentiation (McGuckin et al., 2008). VSELs were isolated from human cord blood also with an improved protocol based on the isolation of enriched progenitor cells by depletion of non-progenitor cells with magnetic separation (Monti et al., 2017). The alternative isolation strategy was a two-step procedure based on the hypotonic lysis of erythrocytes followed by multi-parameter FACS sorting (Halasa et al., 2008). The red cell lysis was probably crucial to the strategy success. VSELs were discarded in the red blood cell fraction by Ficoll-Paque density gradient centrifugation during the processing of bone marrow and cord blood. Instead, when red blood cells were removed by Hespan sedimentation, CD45 (−)/Lin(−)/SSEA-4 (+) VSELs were mostly recovered in the final product (Chang et al., 2014).

More than a thousand genes are downregulated in VSELs, as well as many membrane receptors, cells signaling molecules and CDKs mRNAs. A discordance exists with embryonic stem cells in the expression levels of some pluripotent genes, which could explain some differences in the properties of the two stem cell populations (Lahlil et al., 2018). VSELs from the cord blood, cultured with retinoic acid formed dense colonies and cystic embryoid bodies and differentiated toward the three germ layer lineages as shown by the positivity to specific markers. VSEL differentiation toward mesodermal lineages was also shown upon exposure to an *in vitro* inductive protocol, which promoted the acquisition of a renal progenitor cell phenotype (Monti et al., 2017). DNA analysis and cell cycle studies revealed that the majority of VSELs were diploid, non-apoptotic and in G0/G1 phase, reflecting their quiescent state (Shaikh et al., 2015).

The capacity of VSELs to expand and differentiate *in vitro* in appropriate media was evaluated by Lahlil et al. (2018). After 12 days culture VSELs were significantly expanded for the first time without feeder cells and maintaining their differentiation potential. This makes them possible candidates for regenerative medicine. However, the *in vitro* expansion of VSELs present some critical issues and this for some labs and clinics could result in a limitation for their adoption for clinical purposes. Green fluorescent labeled bone derived VSELs injected in the tail vein of mice after acute liver injury were detected in the liver parenchyma (Nakatsuka et al., 2016). Mouse bone-marrow VSELs express c-kit, the receptor of the stem cell factor (SCF). *In vitro*, in the presence of the hepatocyte growth factor, SCF promoted VSEL differentiation into hepatic colonies. *In vivo*, transplantation of VSELs directly into CCl4-induced injured livers significantly reduced serum alanine aminotransferase (ALT) and raised aspartate aminotransferase (AST) suggesting that VSELs play a role in the repair of injured livers (Chen et al., 2015) Interestingly, rat bone derived VSELs reduced scar area and improved cardiac function significantly in a rat heart infarct model (Wu et al., 2012). Transplantation of VSELs previously exposed to a hypoxic environment and co-expressing HIF-2*α* and Oct4 caused a greater improvement in cardiac function in the same rat heart infarct model (Zhang et al., 2017). Bone-marrow VSELs have the capability to migrate and localize in an injured spinal cord after transplantation (Golipoor et al., 2016). VSELs from patients with critical limb ischemia (CLI) were lower in bone-marrow (*p* < 0.001) and higher (*p* < 0.001) in peripheral blood compared to healthy controls, suggesting that ischemia may trigger VSEL mobilization in this patient population. In agreement with this finding sorted bone-marrow VSELs triggered post-ischemic revascularization in immunodeficient mice (Guerin et al., 2015). Abouzaripour et al. reported that intravenously implanted labeled VSELs migrated into the pancreas of diabetic mice and survived. In treated animals, blood glucose decreased significantly for at least 2 months (Abouzaripour et al., 2015).

It remains to be clarified the relationship between epiblast-like small stem cells and stem cells of the Muse cell group, including also MAPCPs and MIAMI cells. The two stem cell populations share some properties, such as the expression of pluripotent genes and the potential to differentiate toward cells of lineages derived from all three germ layers, but they have also some diversities, such as a different proliferative attitude when transferred in *in vitro* culture and a different expression of some cell surface antigens. Given the observed diversities, and in particular the differences in the proliferation behavior, one should also contemplate the possibility that, epiblast-like small stem cells, after being freed from their niche, move to a Muse-like cell to be considered an intermediate stage between epiblast-like small stem cells and transit amplifying cells (Cancedda and Mastrogiacomo, 2023).

### Healing cells circulating in the adult peripheral blood (CH cells)

**FIGURE 3:**
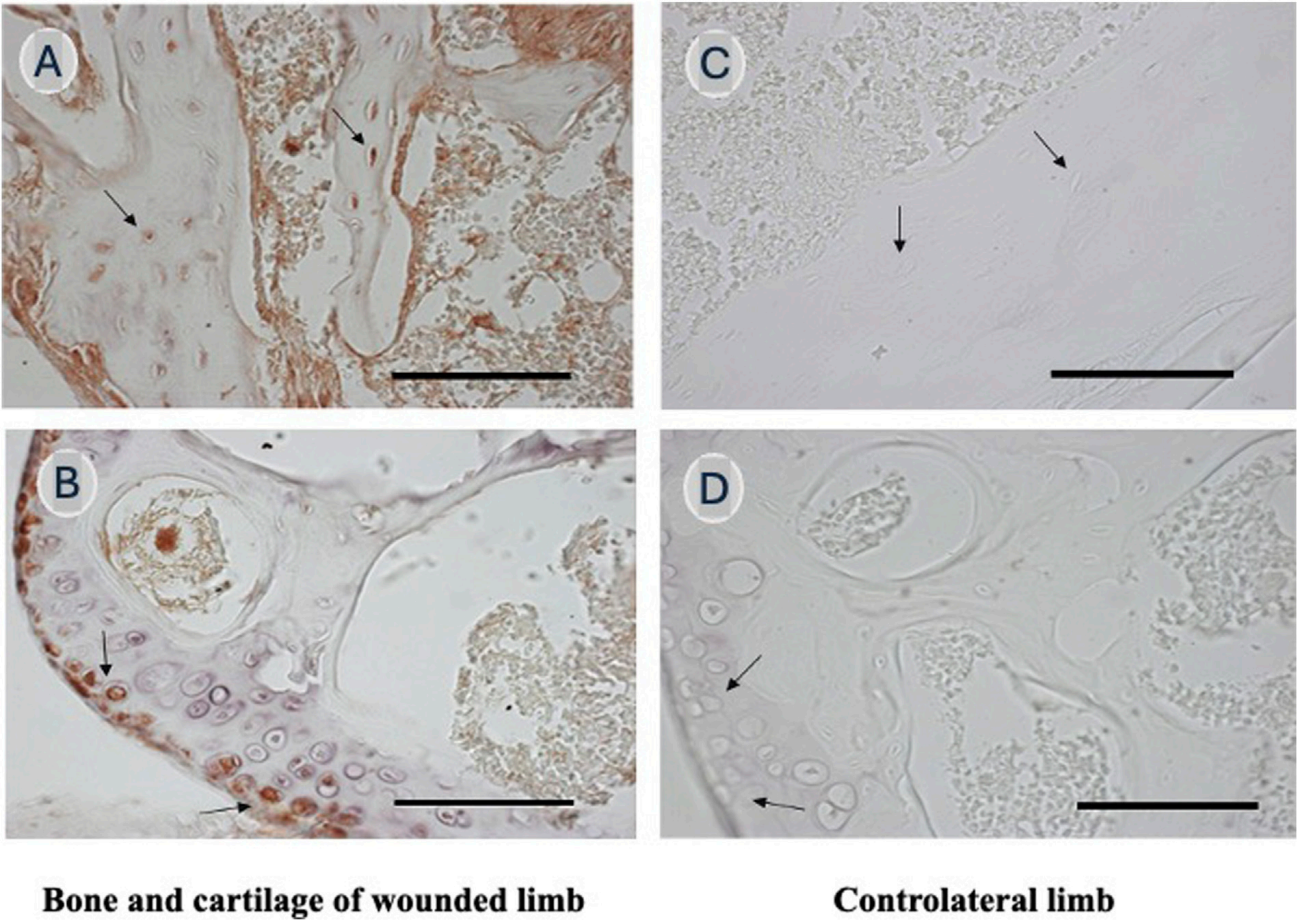
Representative anti-RFP immunostaining of the hard callus and the knee regions from a fractured mouse tail injected with RFP labelled CH cells. Histology sections of the callus formed 24 days after osteotomy **(A)** and of the knee articular cartilage damaged by the insertion of the fixation device **(B)**. The corresponding regions of the contralateral paw of the same mouse are shown in **(C,D)**. Black arrows indicate osteoblasts **(A,C)** and articular chondrocytes **(C,D)**. CH cells homing and differentiation was observed in the damaged tissues but not in the “healthy” paw. Scale bar 100 μm. Modified from Lo Sicco et al. (2015).

Although one can suppose that peripheral blood pluripotent stem cells are also playing a role in tissue homeostasis and cell turnover occurring during tissue life, there is a lack of consensus on the existence of circulating pluripotent stem cells in physiological conditions. Thanks to an innovative flow-cytometry strategy, Lo Sicco et al. identified and isolated a rare cell population involved in the healing processes (CH cells), but also present in the peripheral blood of healthy mice. These circulating cells are characterized by a very small size (less than 4 micron diameter), a poor cytoplasm content, and do not express the pan-hemopoietic CD45 and lineage markers (Lo Sicco et al., 2015). Their transcriptome profile revealed a uniqueness when compared to other cells characterized by varying stemness degree, and a high expression of key pluripotency- and epiblast-associated genes. Red labeled CH cells derived from healthy Red Fluorescent Protein (RFP)-transgenic mice, when blood injected into syngeneic fractured wild-type mice, migrated, and engrafted in the wounded tissues and eventually differentiated into tissue-specific cells (Figure 3). The cell engraftment in the wounded tissues was accompanied by a reduction in the number of CH cells circulating in the peripheral blood.

In a subsequent article Lo Sicco et al. identified BST2 (bone marrow stromal cell antigen 2, also called CD317), a lipid raft-associated integral membrane protein, as a CH cell marker that allowed their identification and defined their bone marrow (BM) compartmental origin (Lo Sicco et al., 2018). BM-derived BST2 pos, Lin neg, CD45 neg cells co-expressed the leptin-receptor (LepR) cell surface antigen, hallmark of a subpopulation of quiescent adult bone-forming Mesenchymal Stem Cells. In response to injury environmental signals, BST2-expressing CH cells: i) proliferated; ii) modified the expression profile of specific cell motility-associated genes and migrated toward the damaged site; iii) responded to the systemic factor Hepatocyte Growth Factor Activator (HGFA), a stimulus inducing the transition of quiescent progenitor cells into the G_ALERT_ state described by (Rodgers et al., 2017).

### Do gonads contain pluripotent cells?

The fusion of female and male gametes can give rise to a new individual of the same species. Oocytes are formed at a relatively early stage of development, whereas spermatozoa are continuously produced during the whole life. Therefore, the question is: are there pluripotent stem cells in gonads, especially in men, from which gametes are derived? Mouse and human spermatogonia are a subclass of undifferentiated male germ cells that give rise to spermatocytes (Kanatsu-Shinohara and Shinohara, 2013). In turn, Spermatogonia Stem Cells (SSCs) are a subpopulation of spermatogonia located at the basement membrane of the seminiferous tubules, where they play a crucial role in spermatogenesis which generate billions of haploid spermatozoa daily. Gametes themselves are not pluripotent whereas the zygote, formed by the gametes, is totipotent. SSCs can be considered pluripotent since they can differentiate into cell lineages from all three germ cell layers. Seandel et al. isolated from adult mammalian testis Multi-potent Adult Spermatogonia Stem Cells (MASCs) with properties like Muse cells from bone marrow. Highly proliferative adult spermatogonia progenitor cells (SPCs) were efficiently obtained by cultivation on mitotically inactivated testicular feeders containing CD34^+^ stromal cells. SPCs exhibit testicular repopulating activity *in vivo* and maintain the ability in long-term culture to give rise to multi-potent adult spermatogonia-derived stem cells (MASCs) (Seandel et al., 2007). The definition of a marker profile for SSCs is difficult since differences exist between different species. GPR125, CD49f, PLZF, UCHL1, GFRA1 and THY1 are expressed in both human and rodent SSCs, but human SSCs do not express some markers, such as Oct-4 and KIT, which are expressed by rodent spermatogonia and SSCs (Chen et al., 2020). For the maintenance of the self-renewal capacity of the SSCs a crucial role is played by the interaction with the microenvironment in where the cells are located. The SSCs “niche” is formed by the surrounding cells and tissues, including Sertoli cells, peritubular myoid cells and testis mesenchyme, and by cytokines and factors such as GDNF, LIF, FGF2, CXCL12 secreted by the “niche” cells and whose receptors are present on SSC surface (Chen et al., 2020).

### Pluripotent stem cells and aging

A decline in the differentiation efficiency both *in vitro* and *in vivo* of cells derived from older donors in both mice and humans have been reported. Aging is the result of a balance between damage and repair (Haigis and Yankner, 2010). With aging, stress responses and restoration pathways, including DNA damage repair networks and mitochondrial respiratory metabolism recovery, progressively decline. Epigenetic modifications are probably responsible of the decline. However, once a cell differentiation process is initiated it remains possible and qualitative.

Relevant mechanisms affecting cell senescence are: i) the accumulation of DNA mutations, ii) telomere shortening, iii) epigenomic modifications, including DNA methylation. These senescence-related nuclear alterations could affect both somatic and stem cells of adult tissues as they age (Shin et al., 2011). In case of stem cells, additional factors triggering a change in the cell status and behavior are the modifications in nature and composition of the “niche” and the resulting mutated interaction of the cells with their immediate microenvironment. It is widely believed that the nuclear alterations and the mutated cell-niche interactions are leading to a decrease in the stem cell self-replication and differentiation potential thus contributing to aging and to age-related diseases.

Comparing the number of VSELs and their pluripotential in normal 4-week-old and 2-year-old mice Ratajczak et al. observed a decrease during ageing (Ratajczak et al., 2010; Ratajczak et al., 2011). VSELs from old mice had a reduced expression of pluripotent genes. Moreover, in the old mice VSELs, the Oct4 promoter was hypermethylated and had a closed chromatin structure. Other changes in the DNA methylation suggested a cell increased sensitivity to the insulin/insulin growth factor (Ins/Igf) signaling that negatively correlates with longevity.

A progressive reduction in the number of VSELs in human peripheral blood depending on the age was evaluated using flow cytometry analysis in healthy volunteers distributed among three groups of age: “young” (mean age, 27.8 years), “middle” (mean age, 49 years), and “older” (mean age, 64.2 years). However the comparison of the three groups, was not revealing any statistically significant difference in the VSELs number, nor differences were observed in mRNA expression for pluripotent markers as SSEA-4, Oct-4, Nanog, and Sox2 (Sovalat et al., 2016). This suggests that the bone-marrow stem cells that remain “healthy” maintain the capacity to be mobilized in the blood stream by the appropriate stimuli from damaged tissues and, possibly, from the physiological cell turnover of tissues.

Although the frequency of MIAMI cells, among all marrow nucleated cells, decreases from 0.01% at age 3% to 0.0018% at age 45, their numbers remained unchanged after age 45. The expression of the markers characteristic of MIAMI cells remained constant independent of age and gender. In long-term *in vitro* cultures, the aging increased the population doubling time by about 30%, whereas the differentiation toward osteoblastic cells was unaffected (D’Ippolito et al., 2006).

## Conclusions: a unifying picture

Despite the many publications existing on the subject, the origin, nature, properties, and even the existence of pluripotent stem cells in mammalian adult tissues and peripheral blood remain very debated and controversial topics. Stem cells derived from adult tissues have been variously described in the literature with diverse names. It is likely that similar or overlapping populations isolated with different experimental strategies were named differently. Moreover, some information was obtained in mice, others in humans and not always the development processes are comparable in the two species, especially regarding the expression of cell surface antigens which are markers of differentiation stages.

**FIGURE 4:**
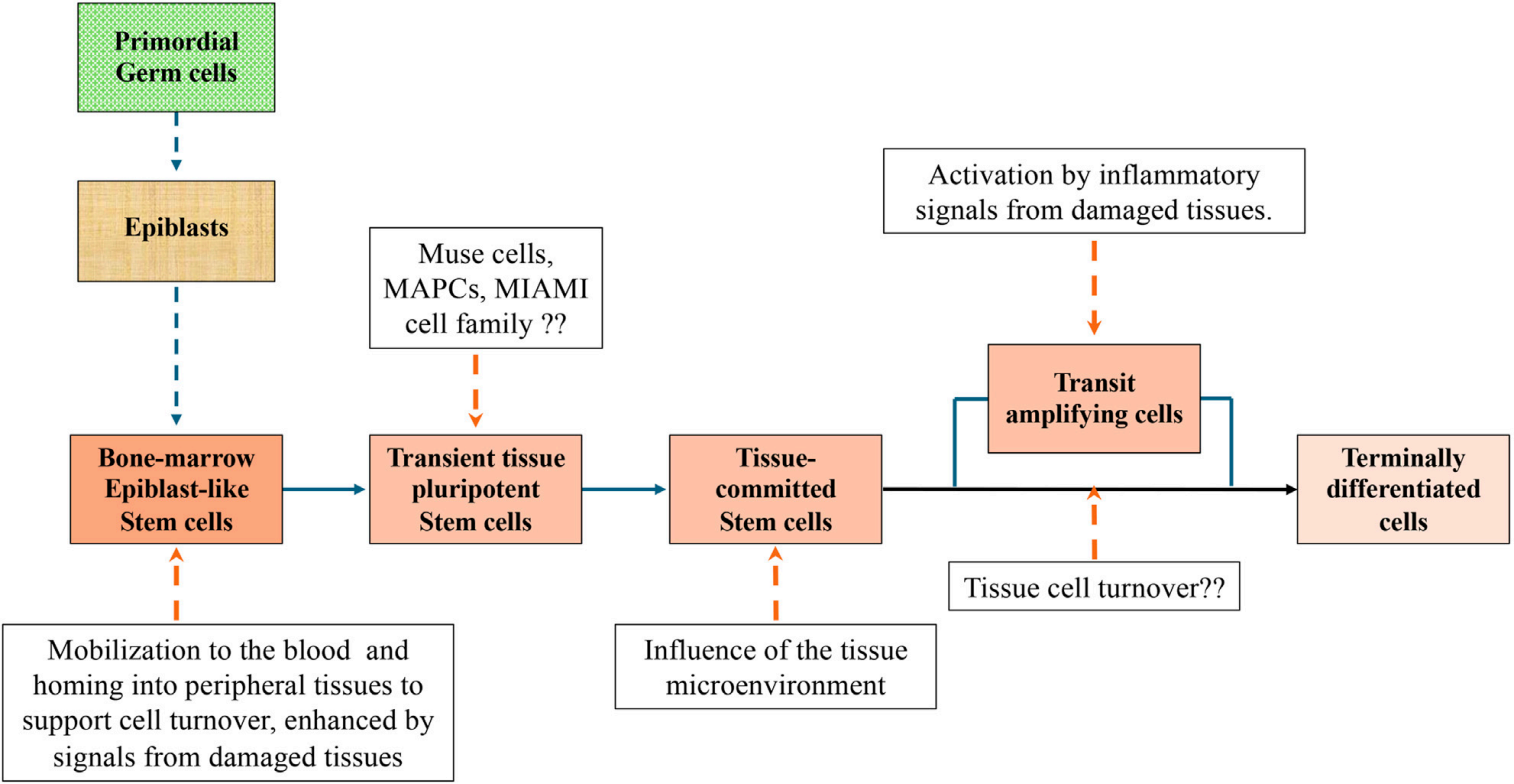
A unifying picture of pluripotent stem cells development to terminally differentiated cells in mammalian adult tissues. The picture is based on contributions to the literature made by different authors. Additional experiments may prove that some parts are wrong or need substantial adjustments. However, we believe that the picture could be a good starting point for future experiments.

In the attempt to reconcile observations made by different authors, we propose a unifying picture (Figure 4):

• In the very early embryos, and possibly also in many fetus tissues, all cells are pluripotent. ES cells, isolated from the inner cell mass of the blastocyst, are the prototype of pluripotent stem cells. At birth, pluripotent cells were often isolated from umbilical cord and cord blood.
• In mammals, after birth and up to adulthood, epiblast-like pluripotent cells are maintained in the bone-marrow and probably in the gonads. They are a very small percentage of the total cell population. These pluripotent cells are characterized by a small size, a scarcity of cytoplasm, a large nucleus, and the expression of cell surface stage specific embryonic antigens (SSEA) and pluripotent genes, expressed in the early embryos such as *Oct3/4, Sox2, Klf4, Nanog*, and *c-Myc.* As ES cells, they have a capacity of self-renewal and the potential to differentiate toward lineages derived from all three germ layers, ectoderm, mesoderm, and endoderm. By all means, they can be classified as adult pluripotent stem cells. However, at variance with ES cells they have lost the teratogenic and tumorigenic properties after their grafting in immunodeficient mice.
• Adult pluripotent stem cells are quiescent and self-renew at very low rate. They are maintained in that state under the influence of the “niche” inside which they are located. The “niche” is constituted by a variety of surrounding cells and by cytokines and other molecules released by these cells and by the stem cells themselves. Mobilization of bone-marrow pluripotent stem cells to the blood stream could result in their homing into peripheral tissues.
• A population of bone-marrow cells with similar, but not identical, properties of the epiblast-like small stem cells, were isolated either directly from the marrow aspirates or from populations of cultured bone-marrow stromal cells (MSCs). More rarely pluripotent cells were isolated also from peripheral tissues other than bone-marrow. These cells were variously described and named in the literature (Muse cells, MAPCs, MIAMI, etc.). However, it is likely that all authors referred to the same cell population and that diversities observed were the results of differences in the protocols adopted for cell isolation and expansion. Outside their “niche” these pluripotent stem cells in the proper culture medium could be expanded as monolayers and maintained for the first culture passages several of the initial properties, including the capacity to give rise to cells of tissues derived from the three germ layers. With the increased number of doublings, they tend to move to the stage of Transit Amplifying Cells and eventually to become senescent or differentiated cells depending on the contest where they are located. It remains to be investigated whether epiblast-like small pluripotent cells appearing early during embryogenesis should be considerate equivalent to the pluripotent stem cells of the Muse group or if Muse cells are an intermediate stage between epiblast-like small stem cells and transit amplifying cells.
• Any tissue damage causes an inflammatory response and the release in the blood circulation of cytokines and molecules that activate the bone-of marrow stem cells and their mobilization. Mobilization of stem cells can be experimentally induced by the administration of G-CSF alone or combined with an inhibitor of the SDF-1 binding. Mobilized stem cells home in the damaged tissue and participate in the repair process. As alternative, in some tissues, the inflammatory response also determines the dedifferentiation of already differentiated cells and their reversion to a progenitor stage.
• There is evidence that from some adult tissues can be isolated cells expressing stage specific embryonic antigens and pluripotent transcription factors such as Oct-4 and Nanog, maintaining a differentiation potential toward more than one germ layer lineage. It has been suggested that they are populations of pluripotent stem cells deposited in embryogenesis during early gastrulation. We prefer to think that they are pluripotent stem cells migrated from the peripheral blood into the tissues in response to a local stimulus. It remains to be investigated if pluripotent stem cells can be mobilized and play a role also in the physiological cell turnover occurring in tissues, especially the ones with a continuous renewal of cells such as blood and skin.
• Following their integration in the microenvironment of the new tissues, the stem cells lose the pluripotency and convert to multi or unipotent stem cells capable to give rise only to the tissue specific cells or, at most, to cells of tissues with the same germ layer origin. Stem cells with a restricted differentiation capability have been isolated from several tissues, also including the isolation of hemopoietic stem cells from the bone-marrow. Very often tissue resident stem cells were isolated from adipose tissue. Although there are no real proofs for this, it is suggestive to think that, because of its chronic inflammatory attitude, adipose tissue is particularly attractive for circulating stem cells.

Additional experiments may prove that some of our statements are wrong or need substantial adjustments. However, we believe that the proposed picture represents a good starting point for future experiments.
